# GALNT3 Inhibits the Progression of Cerebral Ischemia–Reperfusion Injury by Stabilizing TREM2 via O‐GalNAc Glycosylation

**DOI:** 10.1002/cns.70828

**Published:** 2026-03-11

**Authors:** Xinyi Fei, Jinghui Yang, Rui Zhang, Yu Zhang, Shan Yu

**Affiliations:** ^1^ Department of Neurology China‐Japan Union Hospital of Jilin University Changchun Jilin China; ^2^ Department of Hepatobiliary and Pancreatic Surgery China‐Japan Union Hospital of Jilin University Changchun Jilin China

**Keywords:** cerebral ischemia–reperfusion injury, GALNT3, M1 microglia, O‐glycosylation, TREM2

## Abstract

**Background and Objective:**

Cerebral ischemia–reperfusion injury (CIRI) is a major cause of poor outcome after ischemic stroke, and effective therapeutic targets are lacking. This study aimed to investigate the role of polypeptide N‐acetylgalactosaminyltransferase 3 (GALNT3) in CIRI.

**Methods:**

The mouse transient middle cerebral artery occlusion/reperfusion (tMCAO/R) model was established, and transcriptomic analysis of the peri‐infarct cortex was performed to screen target genes. In vivo experiments, neurological scoring and TTC staining were used to assess the effects of GALNT3 on brain injury. In vitro, microglial HMC3 cells subjected to oxygen–glucose deprivation/reoxygenation (OGD/R) were used to model CIRI and further analyze the effects of GALNT3 on macrophage polarization and inflammatory factor expression.

**Results:**

Transcriptomic analysis of the peri‐infarct cortex in the tMCAO/R model identified GALNT3 as a significantly down‐regulated gene. GALNT3 overexpression reduced infarct volume, improved neurological function, and suppressed neuronal apoptosis, oxidative stress, and neuroinflammation in tMCAO/R mice. Subsequently, GALNT3 was further demonstrated to inhibit microglial M1 polarization and down‐regulate inflammatory factors in the OGD/R model. Further mechanism investigation revealed that GALNT3, acting as a mucin type O glycosylation initiating enzyme, directly interacted with triggering receptor expressed on myeloid cells 2 (TREM2) and promoted its O GalNAc glycosylation, predominantly at serine 147, thereby enhancing TREM2 protein stability. TREM2 knockdown stimulated inflammatory responses that were inhibited by GALNT3 overexpression in OGD/R‐treated microglia.

**Conclusion:**

This study revealed a novel GALNT3/TREM2 regulatory axis that restricts neuroinflammation and injury after CIRI, highlighting GALNT3‐mediated O‐glycosylation as a potential therapeutic strategy for CIRI.

## Graphical abstract

The neuroprotective effect of GALNT3 against CIRI and the underlying mechanisms. GALNT3 directly interacts with and catalyzes the mucin‐type O‐GalNAcylation of TREM2 predominantly at serine 147, which enhances TREM2 protein stability. This GALNT3‐TREM2 axis suppresses the release of pro‐inflammatory cytokines, oxidative stress, and activation of M1 microglia. Together, these coordinated actions ultimately relieve CIRI.

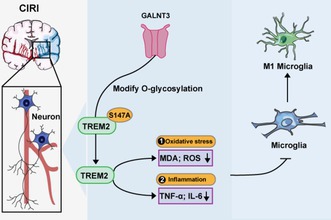

## 1 Introduction

Ischemic stroke is an important cause of severe disability and death for patients worldwide and constitutes a serious socioeconomic burden [1]. At present, the globally recognized treatment strategy is rapid blood flow reconstruction, including intravenous thrombolysis with recombinant tissue plasminogen activator and mechanical thrombolysis through intravascular intervention [2]. However, although some patients are treated promptly and achieve blood flow recirculation, the brain tissue still suffers irreversible damage, even more severe than expected damage; cerebral ischemia–reperfusion injury (CIRI) is the main cause of this condition [3].

The pathogenesis of CIRI is complex, involving a series of complex pathophysiological processes, including inflammation, oxidative stress, apoptosis, and ferroptosis [4, 5]. The microglia in the inflammatory immune response have gained widespread attention in recent years. Classically activated M1 microglia can cause neuronal dysfunction, injury, and degeneration, have strong phagocytosis and cytotoxicity, and promote the secretion of IL‐1β, IL‐6, IFN‐γ, and other inflammatory mediators as well as iNOS and ROS after polarization [6]. In addition, the role of oxidative stress in the pathogenesis of CIRI should not be ignored. Restoring blood supply to ischemic tissue, while restoring aerobic metabolism, also leads to the production of ROS, which exceeds the ability of brain tissue to neutralize ROS and leads to oxidative stress [7]. Many factors intermingle with each other in multiple aspects of the development of CIRI and are causative of each other, ultimately leading to neuronal cell death in brain tissue. Currently, no effective therapeutic measures have been established to prevent further damage caused by reperfusion.

It is well known that glycosylation plays a key role in the post‐translational modification of proteins and lipids, which can regulate the stability and activity of biomolecules. According to the chemical bonds formed between amino acid residues and sugars, glycosylation modifications can be divided into N‐glycosylation and O‐glycosylation [8, 9]. Among them, O‐GalNAcylation is one of the most common O‐glycosylation modifications, commonly found in membrane proteins and secreted proteins [10]. The initial step of O‐GalNAcylation is generally catalyzed by members of the acetylgalactosamine family [11]. Polypeptide N‐acetylgalactosaminyltransferase 3 (GALNT3, also known as GalNAc‐T3 and HFTC) is a member of the acetylgalactosamine family that transfers N‐acetylgalactosamine to the hydroxyl group of serine or threonine residue forming the Tn antigen. In addition, Guo et al. found that overexpression of GALNT3 inhibited hypoxia‐induced apoptosis in human umbilical vein endothelial cells and thereby alleviated endothelial cell injury [12]. In published studies, GALNT3 has been found to relieve vascular calcification and inhibit the secretion of inflammatory factors [13]. GALNT3 inhibits virus‐induced activation of NFκB (p65) in A549 cells, and activation of NFκB mediates M1‐type transformation in microglia in CIRI [14, 15]. The above reports suggested the potential of GALNT3 in the direction of inflammation and microglia polarization. In our mRNA‐seq results in transient middle cerebral artery occlusion/reperfusion (tMCAO/R) mice, GALNT3 expression was downregulated in the peri‐infarct cortex. Taken together, we hypothesized that GALNT3 may be involved in CIRI progression.

In the present study, we comprehensively evaluated the role of GALNT3 in CIRI and its possible molecular mechanisms by establishing in vivo tMCAO/R treated animal models and in vitro oxygen–glucose deprivation/reoxygenation (OGD/R)–induced cell models.

## 2 Materials and Methods

### 2.1 Lentivirus Infection

Lentivirus‐mediated GALNT3 overexpression or TREM2 knockdown was conducted as previously described [4]. In brief, the lentiviral vectors were constructed using the shuttle plasmids pLVX‐IRES‐puro (Fenghui Biology, China) for overexpression and pLVX‐shRNA1 (Fenghui Biology, China) for knockdown. For overexpression, the mouse GALNT3 (NM_015736) or human GALNT3 (NM_004482) coding sequences were cloned into the XhoI/NotI sites. For knockdown, the shRNA targeting human TREM2 (NM_018965) was inserted into the BamHI/EcoRI sites (target sequences for TREM2‐shRNA‐1 and TREM2‐shRNA‐2 are 5‐CTCACCATTACGCTGCGGAAT‐3′ and 5‐ATTCCGCAGCGTAATGGTGAG‐3′, and the scramble shRNA targeting 5‐TTCTCCGAACGTGTCACGT‐3′ was used as a control). Lentiviral particles were produced by co‐transfecting HEK293T cells (Cellverse, China) cultured in DMEM supplemented with 10% FBS at 60%–70% confluence. Transfection was performed using Lipofectamine 3000 (Invitrogen, USA) with 14 μg of shuttle plasmid, 10.5 μg of packaging plasmid pSPAX2 (Fenghui Biology, China), and 3.5 μg of envelope plasmid pMD2.G (Fenghui Biology, China). The viral supernatants were harvested at 48 and 72 h post‐transfection, filtered through 0.45 μm filters. Viral titers were determined as follows: Lv‐Vec, 1.8 × 10^8^ TU/mL; mouse Lv‐GALNT3, 1.5 × 10^8^ TU/mL; human Lv‐GALNT3, 1.1 × 10^8^ TU/mL; Lv‐shNC, 1.9 × 10^8^ TU/mL; Lv‐shTREM2, 1.2 × 10^8^ TU/mL. For HMC‐3 cells, a multiplicity of infection (MOI) of 40 was used.

### 2.2 Animals and Transient Middle Cerebral Artery Occlusion/Reperfusion (tMCAO/R)

Male C57BL/6 mice (8 weeks old) were housed and maintained under a 12‐h light/dark cycle with free access to food and water. All animal experiments were followed by the Ethics Committee of China‐Japan Union Hospital of Jilin University (Approval No. 2025383).

Experiment 1: The tMCAO protocol was performed following a recent report [16]. Briefly, the mouse was anesthetized, after which the skin in the neck was cut open, and a nylon suture was inserted into the internal carotid artery along the distal end of the external carotid artery. The artery was occluded for 60 min and the line was withdrawn to allow reperfusion for 24 h. The ischemic penumbra region was collected for mRNA‐seq analysis.

Experiment 2: The mice were randomly divided into four groups: Sham, tMCAO/R, tMCAO/*R* + Vecor, and tMCAO/*R* + GALNT3. One week before tMCAO/R, a total of 1 μL GALNT3 overexpressing or control lentiviruses (1 × 10^8^ TU/mL) were injected into 3 sites of ipsilateral cortex tissues. The location of the injection was as previously described [16]. The coordinates were shown as follows: point 1: 0.3 mm anterior to bregma, 3.0 mm lateral to bregma and 2.0 mm below the skull surface; point 2: 0.8 mm posterior to bregma, 3.0 mm lateral to bregma and 2.0 mm below the skull surface; point 3: 1.9 mm posterior to bregma, 3.0 mm lateral to bregma and 2.0 mm below the skull surface. One week later, mice underwent tMCAO/R surgery.

### 2.3 Verification of Model Establishment

Twenty‐four hours after the operation, the neurological function of animals was scored using the Longa scoring criteria [17]. The brain water content was further measured as reported [17]. The wet weight of the brains was first quantified. Then the brain tissue was dried in an oven at 100°C for 24 h. Cerebral edema was counted according to the formula: (wet weight − dried weight)/wet weight × 100%.

### 2.4 Cell Culture

The human microglia HMC3 cells were obtained from the Cellverse company (China). Cells were cultured with DMEM (Servicebio, China) containing 10% FBS (Tianhangbio, China) at 37°C with 5% CO_2_. Before oxygen–glucose deprivation/reoxygenation (OGD/R) modeling, the medium was changed to DMEM medium containing 5% FBS, and the experiment was conducted one day later.

For the OGD/R model, cells were cultured in RPMI 1640 medium (Solarbio, China) without FBS and placed in the chamber with 100% inert nitrogen gas until the oxygen concentration reached < 1% for 4 h. Cells were then removed from the anaerobic chamber and replaced with the DMEM medium containing 10% FBS in a normoxic environment (5% CO_2_) for 24 h.

### 2.5 Co‐IP

Co‐IP experiments were performed as described in the previous study [18]. Cell lysate was centrifuged at 10000 g for 5 min at 4°C and the resulting supernatant was collected. The samples were incubated with the target IP antibodies for 2 h at room temperature and then added to the corresponding resin (Thermo Fisher, USA) that had cured the corresponding antibodies for 2 h. After incubation, immune complexes were isolated by centrifugation and washed with washing buffer. Finally, the co‐IP was subjected to blot for further analysis using specific antibodies. The primary antibodies including TREM2 antibody (1:500, Santa Cruz, USA), Flag antibody (1:1000, ABclonal, China), HA antibody (1:1000, Affinity, China), Biotin‐VVL (1:3000, Vector Laboratories, China). The secondary antibodies including goat anti‐rabbit (1:3000, Solarbio, China) or goat anti‐mouse (1:3000, Solarbio, China).

### 2.6 *Vicia villosa* Lectin (VVL) Assay

In brief, the Tn antigen in glycoprotein was detected using Biotin‐VVL (Vector Laboratories, USA) agarose beads (Thermo Fisher Scientific, USA). Cell lysate protein (600 μg) was incubated with 4 μg biotinylated lectin at 4°C for 3 h and then incubated with 20 μL streptavidin‐agarose at 4°C for 2 h. The lectin/glycoprotein complex was collected by centrifugation (1400 rpm, 5 min). The protein was precipitated for western blot.

### 2.7 Bioinformatics Analysis

Potential interacting partners of human GALNT3 were predicted using the BioGRID database (https://thebiogrid.org). The search was performed using ‘GALNT3’ as the query gene with *Homo sapiens* as the target organism. The physical interactions from all available evidence types were included. The resulting interaction network, comprising 26 candidate proteins, was extracted for further analysis.

### 2.8 mRNA Squencing

Using the tMCAO/R model, focal cerebral ischemia was induced in male C57BL/6J mice by occluding the right external carotid artery for 60 min, followed by 24 h of reperfusion. Brain tissues from the ischemic penumbra of the ipsilateral cortex were collected from two groups: sham group (*n* = 6) and tMCAO/R group (*n* = 6). Total RNA was extracted and the RNA integrity was assessed using the Agilent 2100 Bioanalyzer. Following qualification, eukaryotic mRNA was enriched using oligo(dT)‐attached magnetic beads. The enriched mRNA was fragmented, and first‐strand cDNA was synthesized using random hexamer primers, followed by second‐strand cDNA synthesis. Library quality was assessed based on fragment size distribution and effective concentration. Qualified libraries were pooled and sequenced on an Illumina NovaSeq 6000 platform to generate paired‐end reads. The clean reads were then aligned to the reference genome (GRCm39) using HISAT2 (v2.2.1). Gene expression levels were quantified as FPKM using HTSeq (v2.0.2). Differential expression analysis between the Sham and tMCAO/R groups was performed using DESeq2 (v1.24.0). Genes with log2FC < −2 and adjp < 0.01 were considered significantly differentially expressed. The mRNA‐seq data is available in GEO under accession number GSE313863.

### 2.9 Statistical Analysis

Statistical analyses were performed via GraphPad Prism 9.5 software. All data were expressed as the mean ± SD. For animal studies: A sample size of *n* = 6 was used per group. All behavioral scoring and pathological assessments were performed by investigators who were blinded to the group allocations. For cell‐based experiments: Data are presented from *n* = 3 independent replicates per group. A two‐tailed student's *t*‐test was used to analyze the difference between two groups, and One‐way ANOVA with Tukey post hoc test was used among multiple groups. The non‐parametric data are expressed as median, and the difference was compared through the Kruskal–Wallis test. ns, nonsignificant, *p < 0.05, **p < 0.01, ***p < 0.001, or ****p < 0.001.

## 3 Results

### 3.1 Identification of Differentially Expressed Genes (DEGs) in the mRNA Seq Results

**FIGURE 1:**
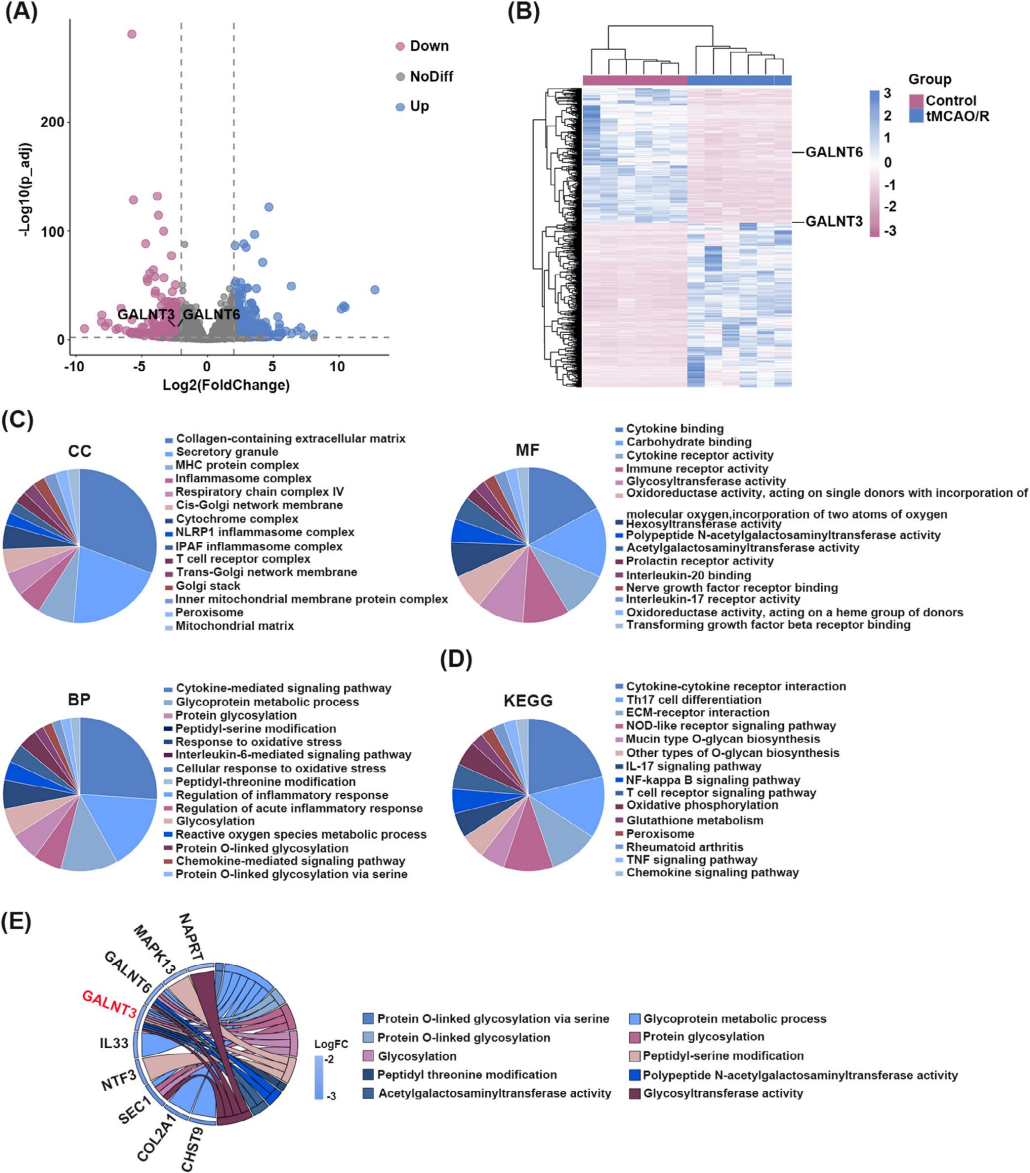
Identification of differentially expressed genes (DEGs) in the mRNA seq results. (A) Volcano plots of DEGs in Sham and tMCAO/R samples in mRNA seq results. DEGs were selected by adjp < 0.01 and log_2_FC > 2 or log2FC < −2. (B) Clustering heatmap of significantly changed DEGs. (C) The GO enrichment analysis of down‐regulated DEGs in three categories (Biological process, Molecular function, and Cell component). (D) The KEGG enrichment analysis of down‐regulated DEGs. (E) The chord diagram showed the DEGs enriched in GO terms. FC, fold change; GO, gene ontology; KEGG, kyoto encyclopedia of genes and genomes.

To investigate transcriptomic changes in cerebral ischemia–reperfusion injury (CIRI), we first performed mRNA sequencing on brain tissues from Sham and tMCAO/R mice. The differential expression analysis (adj *p* < 0.01, |log2FC| > 2) identified a set of significantly dysregulated genes, which are displayed in the volcano plot and a clustering heatmap (Figure 1A,B). In this study, we mainly focused on differentially expressed down‐regulated genes. As shown in Figure 1C, the genes with decreased expression were generally enriched in the collagen‐containing extracellular matrix, secretory granule, and were closely related to biological processes or molecular functions such as cytokine‐mediated signaling pathway, glycoprotein metabolic process, cytokine binding, and carbohydrate binding. In addition, KEGG analysis showed that these genes were significantly associated with 15 pathways including ECM‐receptor interaction and cytokine‐cytokine receptor interaction (Figure 1D). It is well known that the inflammatory response is a central pathological event in CIRI [19]. Post‐ischemic inflammation rapidly activates microglia, and particularly M1 polarization exacerbates neuronal injury and promotes CIRI progression [20, 21, 22]. O‐glycosylation is one of the most common post‐translational modifications, regulating protein stability and participating in inflammatory regulation [23, 24]. Therefore, our research focus lies at the intersection of glycosylation and CIRI. O‐GalNAcylation, one of the most prevalent O‐glycosylation modifications, is initiated by members of the GALNT family [10, 11]. As shown in Figure 1E, in the pathways related to glycosylation, GALNT3 and GALNT6 are significantly enriched. Using the criteria for screening differentially expressed genes, we found that both GALNT3 and GALNT6 were significantly downregulated in the peri‐infarct cortex of tMCAO/R mice. Given that GALNT3 showed a slightly greater degree of downregulation, and it was reported to have anti‐inflammatory effects and to inhibit microglial polarization [13, 14, 15]. Therefore, we prioritized the functional exploration of GALNT3 and hypothesized that GALNT3 might play a role in improving neural injury in CIRI.

### 3.2 GALNT3 Helps Ameliorate Neurological Impairment in tMCAO/R‐Treated Mice

**FIGURE 2:**
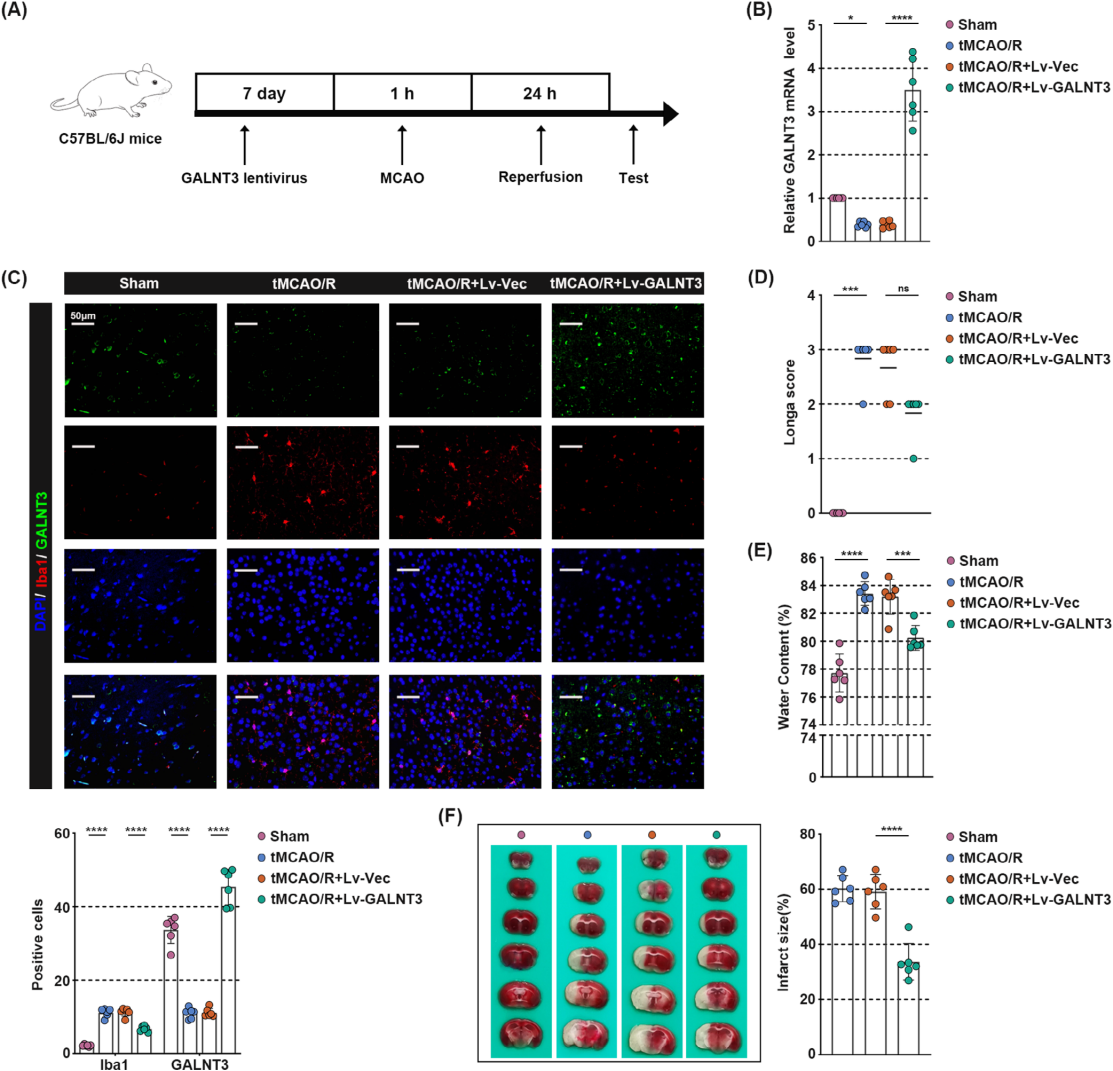
GALNT3 helps ameliorate neurological impairment in tMCAO/R‐treated mice. (A) The schematic diagram of the tMCAO/R model experimental design. (B) The relative mRNA level of GALNT3 in the peri‐infarct cortex was detected by qPCR. (C) The coimmunostaining results of Iba‐1 (red) and GALNT3 (green) in the peri‐infarct cortex. (Scale bar, 50 μm). (D) The Longa score of mouse among different groups. (E) Assessment of brain tissue water content. (F) The results of the TTC staining, the red part represents no infarction and the white part represents infarction (left panel). The percentage histogram of infarct volume among different groups (right panel). Data are shown as mean ± SD. *n* = 6 per group. *p < 0.05, ***p < 0.001, ****p < 0.0001.

To determine whether GALNT3 affects brain injury during CIRI, C57BL/6 mice were injected with GALNT3 overexpressing or control lentivirus in the right ventricle one week before tMCAO/R surgery (Figure 2A). As shown in Figure 2B, compared with the Sham group, GALNT3 was inhibited in the peri‐infarct cortex tissues of tMCAO/R mice, but this phenomenon was reversed by overexpression of GALNT3. In addition, lentivirus‐mediated overexpression of GALNT3 in microglia of tMCAO/R mouse brain tissue was further verified by immunofluorescence double staining (Figure 2C). The results of Longa scoring on the mouse model showed that tMCAO/R led to decreased neurological function, but GALNT3 overexpression partially restored neural function (Figure 2D). Furthermore, we found that tMCAO/R induced more severe brain edema (Figure 2E) and infarct size (Figure 2F). However, GALNT3 overexpression largely reversed these phenomena. Taken together, these results suggested that GALNT3 helped ameliorate neurological impairment in tMCAO/R‐treated mice.

### 3.3 GALNT3 Inhibits Neuronal Apoptosis and Oxidative Stress in tMCAO/R Mice

**FIGURE 3:**
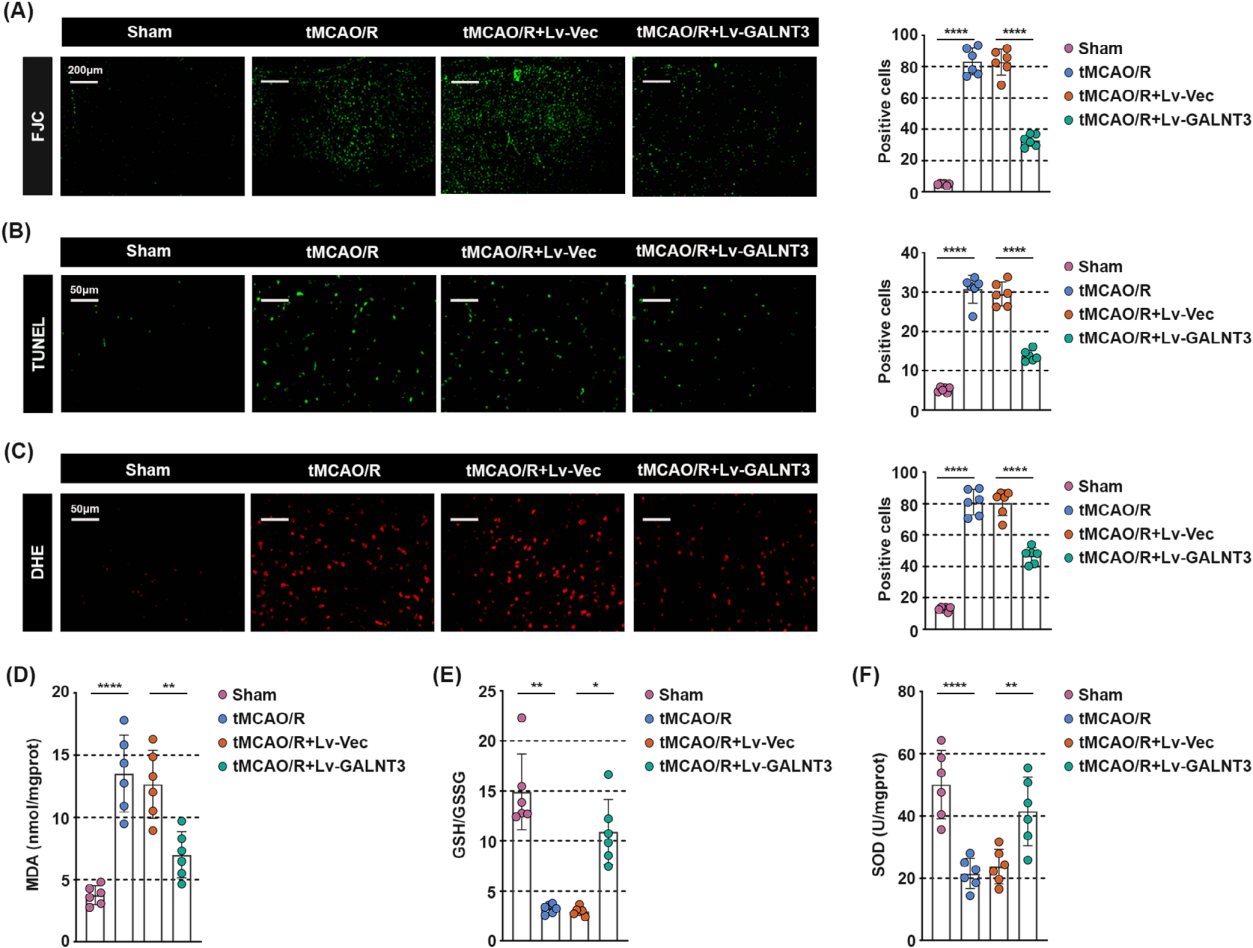
GALNT3 inhibits neuronal apoptosis and oxidative stress in tMCAO/R mice. (A) Degenerating neurons were measured by FJC staining. (Scale bar, 200 μm). (B) The immunostaining results of TUNEL in the peri‐infarct cortex. (Scale bar, 50 μm). (C) The production of ROS was detected by DHE staining. (Scale bar, 50 μm). (D‐F) The level of MDA, GSH/GSSG, and SOD was detected by Kits, respectively. Data are shown as mean ± SD. *n* = 6 per group. *p < 0.05, **p < 0.01, ****p < 0.0001.

We further evaluated the role of GALNT3 on neuronal apoptosis and oxidative stress in tMCAO/R mice. Generally, neuronal degeneration often occurs in the early stages of CIRI [25]. We examined the effect of GALNT3 on neuronal degeneration in the ischemic penumbra of tMCAO/R mice using FJC staining. We found that FJC fluorescence intensity was significantly increased in the tMCAO/R group compared with the sham‐operated group, but significantly decreased after GALNT3 treatment (Figure 3A). In addition, overexpression of GALNT3 inhibits neuronal apoptosis to a certain extent (Figure 3B). Accumulating studies have shown that reperfusion leads to highly deleterious ROS production, generating oxidative stress, which contributes to the majority of ischemia–reperfusion brain injuries [26, 27]. We found by DHE staining that GALNT3 overexpression reversed the overproduction of ROS caused by tMCAO/R treatment (Figure 3C). Our data also showed that malondialdehyde (MDA) accumulation in tMCAO/R mice was positively correlated with the degree of oxidative stress, but GALNT3 significantly suppressed this trend (Figure 3D). In addition, tMCAO/R treatment resulted in a decrease in glutathione (GSH)/oxidized glutathione (GSSG) ratio and superoxide dismutase (SOD) activities, but GALNT3 restored these values (Figure 3E,F). Overall, our data suggested that GALNT3 inhibited neuronal apoptosis and oxidative stress in tMCAO/R mice.

### 3.4 GALNT3 Inhibits Inflammation and Microglial M1 Polarization in tMCAO/R Mice

The role of GALNT3 on inflammation and microglial M1 polarization in CIRI progression was further determined. Specifically, we studied levels of CD16/32, iNOS, TNF‐α, and IL‐6, which are markers of microglia M1 activation and brain tissue inflammation. As shown in Figure S1A, GALNT3 significantly inhibited the increase of CD16/32 fluorescence intensity in the tMCAO/R treated mouse infarction tissue. A similar trend was also seen in the expression of iNOS, another M1‐type microglia marker (Figure S1B). Finally, we examined the effect of GALNT3 on CIRI‐induced inflammation. As shown in Figure S1C, the levels of IL‐6 and TNF‐α were significantly elevated after CIRI injury, but GALNT3 overexpression inhibited the expression of these inflammatory factors. In summary, our results indicated that GALNT3 inhibited inflammation and microglial M1 polarization in tMCAO/R mice.

### 3.5 GALNT3 Inhibits Inflammatory Response and M1 Polarization in Oxygen–Glucose Deprivation/Reoxygenation (OGD/R)‐induced Microglia

**FIGURE 4:**
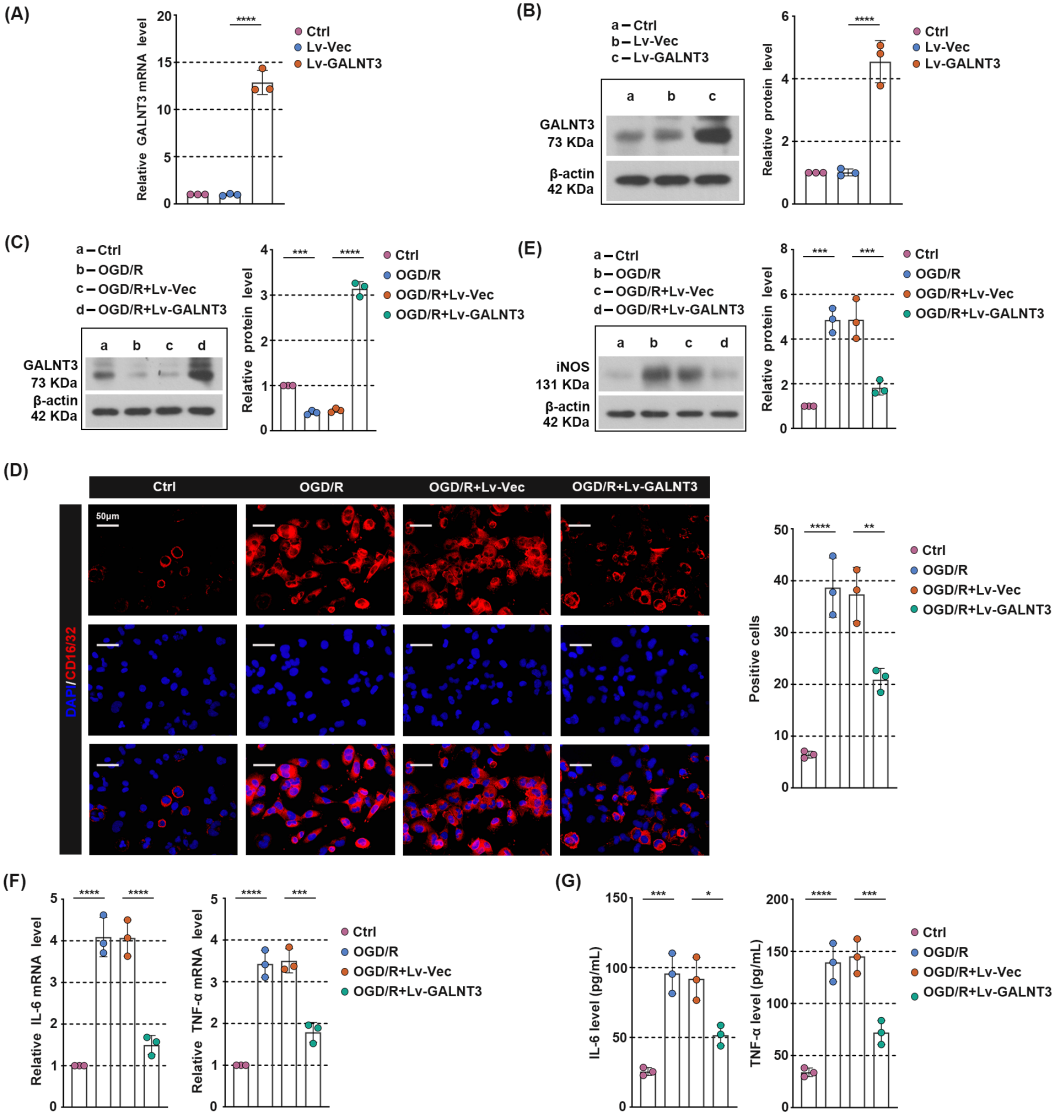
GALNT3 inhibits inflammatory response and M1 polarization in oxygen–glucose deprivation/reoxygenation (OGD/R)‐induced microglia. (A, B) The HMC‐3 cells were infected with GALNT3 overexpression or control lentivirus, and the expression of GALNT3 was detected by qPCR and western blot after 48 h. (C) Western blot analysis of GALNT3 in cells of each group. (D) The immunostaining of CD16/32 in the cells. (Scale bar, 50 μm). (E) Western blot analysis of iNOS in cells of each group. (F) The relative mRNA level of IL‐6 and TNF‐α was detected by qPCR. (G) The level of IL‐6 and TNF‐α in the supernatant was detected by ELISA. Data are shown as mean ± SD. *n* = 3 per group. *p < 0.05, **p < 0.01, ***p < 0.001, ****p < 0.0001.

To further confirm the involvement of GALNT3 in CIRI, we exposed microglia HMC‐3 cells to OGD/R to simulate ischemic injury in vivo. The infection efficiency of GALNT3 overexpressing lentivirus was first verified by qPCR and western blot, respectively (Figure 4A,B). In addition, GALNT3 expression was suppressed in OGD/R‐treated HMC‐3 cells compared with normal cells, but this was reversed by GALNT3 overexpression (Figure 4C). We then evaluated the M1‐polarization and inflammatory response of microglia in vitro. As shown in Figure 4D,E, the expression of CD16/32 and iNOS was significantly up‐regulated in OGD/R‐treated microglia, but was inhibited by the overexpression of GALNT3. In addition, GALNT3 reduced the increase in IL‐6 and TNF‐α levels caused by OGD/R stimulation, which was consistent with the trend reflected in vivo results (Figure 4F,G). To further confirm the functional necessity of GALNT3, we knocked down GALNT3 in HMC‐3 cells using shRNA (Figure S2B). In OGD/R‐treated cells, GALNT3 knockdown further enhanced the expression of the M1 markers CD16/32 and iNOS (Figure S2C–D) and promoted the release of IL‐6 and TNF‐α (Figure S2E), indicating that loss of GALNT3 exacerbates inflammatory responses and M1 polarization. Overall, our results demonstrated that GALNT3 inhibited inflammatory response and M1 polarization in OGD/R‐induced microglia.

### 3.6 GALNT3 Promotes the O‐GalNAc Glycosylation of TREM2 Protein

**FIGURE 5:**
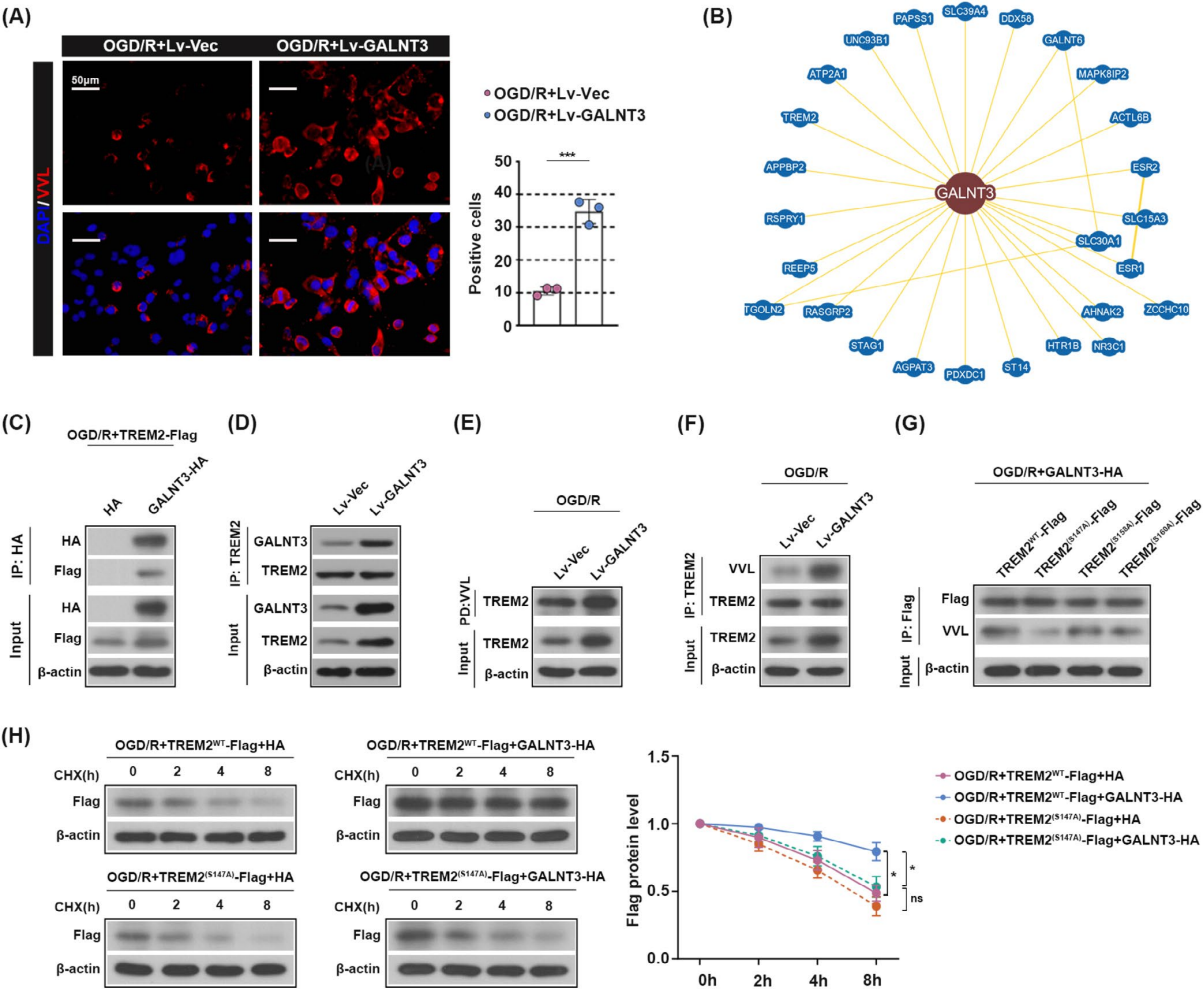
GALNT3 promotes the O‐GalNAc glycosylation of TREM2 protein. (A) The immunostaining of Tn antigen (VVL, red) in the cells. (Scale bar, 50 μm). (B) The PPI network diagram of GALNT3 interacting protein from the BioGRID database. (C) The cells were co‐transfected with the GALNT3‐HA overexpression plasmid and the TREM2‐FLAG overexpression plasmid. After 48 h of transfection, the OGD/R model was established, and Co‐IP was used to detect the binding situation of GALNT3 and TREM2. (D) The interaction between GALNT3 and TREM2 was assessed by Co‐Immunoprecipitation (Co‐IP) using an anti‐TREM2 antibody for immunoprecipitation. (E) The effect of GALNT3 on the O‐GalNAc glycosylation of TREM2 was detected by the VVL pulldown assay. (F) Co‐IP combined with VVL blotting assay to detect whether GALNT3 mediates O‐GalNAc glycosylation of TREM2. (G) HMC‐3 cells were co‐transfected with GALNT3‐HA overexpression plasmid and TREM2^WT^‐Flag or TREM2 ^(S147A, S158A, S160A)^‐Flag overexpression plasmid and the OGD/R model was constructed 48 h later. The O‐glycosylation sites of TREM2 were detected by Co‐IP combined with VVL blotting assay. (H) HMC‐3 cells were co‐transfected with GALNT3‐HA overexpression plasmid and TREM2^WT^‐Flag or TREM2 ^(S147A)^‐Flag overexpression plasmid. After transfection for 48 h, OGD/R model was constructed and incubated with 20 μg/mL Cycloheximide (CHX) for 0, 2, 4, 8 h. The expression of TREM2 in cells was detected by western blot using anti‐Flag antibody. Data are shown as mean ± SD. *n* = 3 per group. *p < 0.05, ***p < 0.001. Co‐IP Co‐Immunoprecipitation; VVL, vicia villosa lectin.

The pathway through which GALNT3 affects CIRI is unknown. It is well known that GALNT3 acts as a mucin‐type O‐glycosylation initiating enzyme that can initiate O‐GalNAcylation modification and thus affect downstream protein stability [28]. In this study, we first examined the occurrence of O‐GalNAcylation modification in microglia. As shown in Figure 5A, GALNT3 overexpression induced the Tn antigen (VVL) expression in OGD/R‐treated cells, indicating increased global O‐GalNAcylation. On this basis, our analysis of the BioGRID database revealed the existence of 26 proteins that may interact with GALNT3 (Figure 5B). Among these factors, Triggering Receptor Expressed on Myeloid cells 2 (TREM2) caught our attention. TREM2 is an immune receptor expressed mainly in microglia and alleviates CIRI progression by inhibiting microglia M1 polarization [29]. Therefore, we hypothesized that GALNT3 may alleviate CIRI progression by promoting TREM2 glycosylation modification and protein stability. To confirm the physical interaction between GALNT3 and TREM2, we co‐transfected HMC‐3 cells with GALNT3‐HA and TREM2‐Flag plasmids and performed co‐immunoprecipitation using an anti‐FLAG antibody. As shown in Figure 5C, GALNT3 was efficiently co‐precipitated with TREM2. We further performed a Co‐IP using the antibody against endogenous TREM2 to validate this interaction under more physiological conditions. Consistent with Figure 5C, GALNT3 was also precipitated by anti‐TREM2 antibody, confirming the specificity and robustness of the GALNT3‐TREM2 interaction (Figure 5D). Since VVL can bind to unmodified O‐GalNAc residues with high affinity, we further assessed whether GALNT3 affects O‐GalNAc glycosylation of TREM2 by VVL pull down experiments. As shown in Figure 5E, GALNT3 promoted the O‐GalNAc glycosylation modification of TREM2. Subsequently, we found that GALNT3‐mediated O‐GalNAc glycosylation of TREM2 promoted the stability of TREM2 protein to a certain extent by Co‐IP combined with VVL blotting (Figure 5F). We further found the presence of 3 O‐GalNAc glycosylation sites in TREM2 by NetOGlyc database prediction, so we generated Flag‐tagged constructs in which each candidate O‐glycosylation site (S147A, S158A, and S160A) was replaced, and then transfected these constructs as well as the Flag‐tagged TREM2^WT^ plasmids into HMC‐3 cells and detected the TREM2 O‐glycosylation sites by immunoprecipitation combined with VVL blotting. As shown in Figure 5G, the VVL expression was most significantly down‐regulated in cells transfected with Flag‐TREM2^(S147A)^, suggesting that S147A may be the O‐glycosylation site of TREM2. After treatment with the protein synthesis inhibitor actinomycin ketone (CHX), we further examined the stability of the TREM2 protein at different time points. We found that overexpression of GALNT3 promoted TREM2 protein stability, but the half‐life of TREM2 protein was shortened in cells transfected with Flag‐TREM2^(S147A)^, suggesting that GALNT3‐mediated O‐glycosylation of TREM2 at the S147A site promotes the protein stability of TREM2 (Figure 5H). In summary, these data suggested that O‐glycosylation at specific sites by GALNT3 is likely to be important for the stabilization of the TREM2 protein.

### 3.7 GALNT3 Mediates M1 Polarization of Microglia by Up‐Regulating the Expression of TREM2 Protein

**FIGURE 6:**
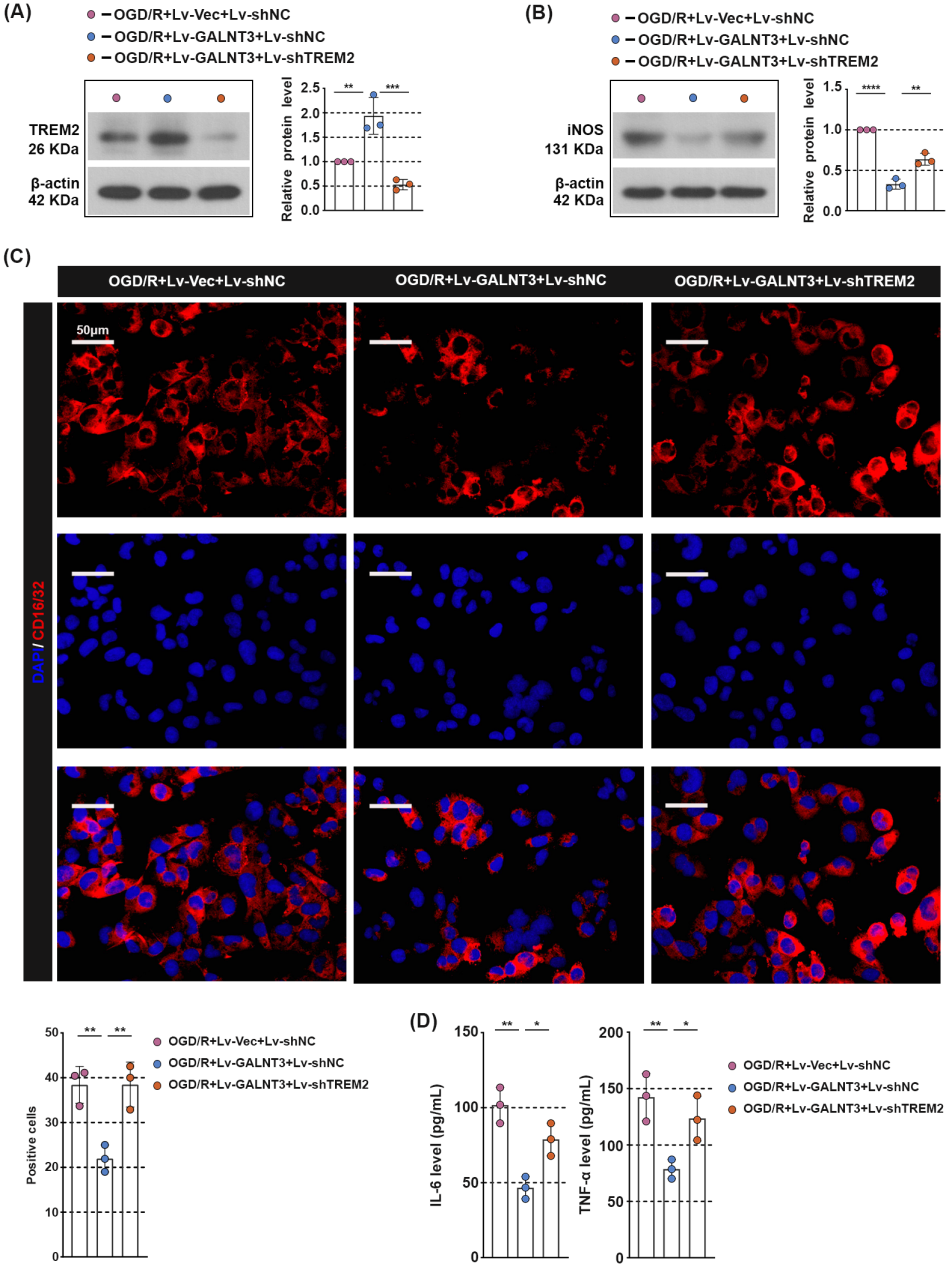
GALNT3 mediates M1 polarization of microglia by up‐regulating the expression of TREM2 protein. (A, B) The HMC‐3 cells were infected with TREM2 knockdown or control lentivirus, and the expression of TREM2 and iNOS was detected by western blot after 48 h. (C) The immunostaining of CD16/32 in the cells. (Scale bar, 50 μm). (D) The level of IL‐6 and TNF‐α in the supernatant was detected by ELISA. Data are shown as mean ± SD. *n* = 3 per group. *p < 0.05, **p < 0.01, ***p < 0.001, ****p < 0.0001.

Finally, we verify the contribution of TREM2 to the action of GALNT3. We first validated the knockdown efficiency of TREM2 in HMC‐3 cells; infection with TREM2‐targeting lentivirus significantly reduced both mRNA and protein levels of TREM2 compared to the control lentivirus (Figure S2A). Next, GALNT3 overexpressing lentivirus and/or TREM3 knockdown lentivirus were co‐infected with HMC‐3 cells, and the expression of TREM2 and iNOS in the cells was detected after OGD/R treatment. As shown in Figure 6A,B, GALNT3 overexpression was accompanied by an increase in TREM2 expression, which also led to the downregulation of iNOS expression, but this trend was reversed by shTREM2. In addition, knockdown TREM2 partially restored the expression of the M1‐type microglia marker CD16/32 caused by GALNT3 overexpression (Figure 6C). Changes in IL‐6 and TNF‐α expression were further evaluated. As shown in Figure 6D, GALNT3 overexpression significantly upregulates the expression of IL‐6 and TNF‐α, but TREM2 knockdown suppresses the expression of these factors. Taken together, these findings suggested that GALNT3 was involved in CIRI progression by mediating TREM2 expression.

## 4 Discussion

In this study, we focused on the neuroprotective effect of GALNT3 against CIRI and its potential mechanisms. Current data suggested that GALNT3 is beneficial to alleviate neurological deficits, inhibit oxidative stress, and the activation of M1 microglia in the course of disease. From the measured concentrations of inflammatory cytokines, we confirmed that GALNT3 treatment significantly inhibited levels of IL‐8 and TNF‐α. Our results showed that GALNT3 may be a novel therapeutic target for CIRI and that this is related to the regulation of TREM2 protein stability.

Generally speaking, reperfusion after vascular recanalization aggravates the destruction of structure and metabolic dysfunction of cells, which further leads to reperfusion injury on top of ischemic injury [30]. This ischemia–reperfusion process leads to a series of pathophysiological changes, including cellular oxidative stress, inflammatory response, and apoptosis, ultimately leading to brain tissue dysfunction and cell death [31]. With all of these events, various molecular changes occur that may affect recovery. To investigate how GALNT3 interferes with CIRI neural function injury and the corresponding mechanism of action, we conducted a series of experiments. In the tMCAO/R experimental model, we observed impaired neurological function, increased infarct area as well as cerebral edema in mice, which is consistent with previous reports [17, 18, 19, 20, 21, 22, 23, 24, 25, 26, 27, 28, 29, 30, 31, 32]. However, mice receiving GALNT3 overexpression treatment showed significant improvement in neurological function, suggesting that GALNT3 is likely to play a neuroprotective role during CIRI.

Oxidative stress plays a crucial role in CIRI, mainly because a large number of ROS are produced during reperfusion, and the imbalance between ROS generation and ROS clearance can lead to aggravated oxidative stress damage of CIRI [33]. Some antioxidants, such as resveratrol and vitamin C, have been found to prevent brain damage in vitro [34, 35]. In addition, accumulating studies have shown increased MDA content and decreased SOD activity in brain tissue of tMCAO/R rats. This suggests the presence of significant oxidative stress in the tMCAO/R model. Our data also confirmed this, and at the same time, we found that GALNT3 can inhibit oxidative stress and regulate MDA and SOD levels in the brain tissue of cerebral ischemic mice. The inflammatory cascade response also occurs during CIRI, when inflammatory cells are activated, releasing inflammatory factors that affect the brain, and activated microglia are the most common manifestations [36, 37]. Microglia are resident immune cells of the central nervous system that continuously monitor the brain microenvironment under normal conditions, and studies have shown that they rapidly activate within minutes of cerebral ischemia. Activated microglia are generally defined as either classically activated (type M1) or alternatively activated (type M2), with type M1 microglia functioning predominantly in the early stages of stroke onset to release proinflammatory factors as a proxy for neurological injury [38, 39]. Our findings are consistent with these studies that the number of M1‐type microglia in the cerebral infarcted tissues of tMCAO/R‐treated mice was proliferated and accompanied by the upregulation of the expression of the pro‐inflammatory factors TNF‐α and IL‐1β. In addition, a similar phenomenon was observed in OGD/R‐treated HMC‐3 cells. These data undoubtedly indicated the positive role of GALNT3 in inhibiting oxidative stress and inflammatory response of M1 microglia.

Next, we focus on the downstream regulatory proteins of GALNT3. TREM2 is an innate immune surface receptor belonging to the immunoglobulin superfamily, which is expressed in the brain mainly in microglia, has been extensively implicated in neuroinflammation and neurodegenerative diseases [40, 41]. TREM2 has been found to play a key role in regulating the inflammatory response and phagocytosis of cellular debris in the central nervous system, thereby exerting neuroprotective effects in various CNS pathologies [42, 43, 44]. In the context of cerebral ischemia–reperfusion injury, Wu et al. reported that TREM2 alleviates neuroinflammation by inhibiting M1 microglial polarization and reducing the release of pro‐inflammatory cytokines such as TNF‐α and IL‐1β [29]. Consistent with these findings, our study confirms that TREM2 plays a protective role in CIRI by restraining microglial activation and inflammatory cascades. Through BioGRID database analysis, we identified a potential interaction between GALNT3 and TREM2, which was subsequently validated by co‐immunoprecipitation assays. Importantly, we provided experimental evidence that GALNT3 catalyzes O‐GalNAcylation of TREM2, predominantly at serine 147, as demonstrated by lectin pulldown and site‐directed mutagenesis. This modification significantly enhances TREM2 protein stability, as evidenced by prolonged half‐life in the presence of GALNT3 overexpression, whereas mutation of the S147 site accelerates TREM2 degradation. To our knowledge, this is the first report linking GALNT3‐mediated O‐glycosylation to TREM2 stabilization, offering a mechanistic explanation for how TREM2 protein levels may be regulated under ischemic stress. Finally, we found that positive regulation of TREM2 by GALNT3 inhibited the M1‐type polarization and inflammatory response of microglia after OGD/R stimulation by performing a series of rescue experiments, which provided sufficient evidence for us to rationalize that GALNT3 exerts neuroprotective effects in CIRI. Importantly, we further investigated the downstream signaling mechanism through which the GALNT3/TREM2 axis exerts its anti‐inflammatory effects. Existing literature indicates that TREM2 inhibits microglial inflammation by suppressing the PI3K/NF‐κB pathway, and GALNT3 has similarly been shown to attenuate vascular inflammation via blocking TNFR1/NF‐κB signaling [13, 14, 15, 16, 17, 18, 19, 20, 21, 22, 23, 24, 25, 26, 27, 28, 29, 30, 31, 32, 33, 34, 35, 36, 37, 38, 39, 40, 41, 42, 43, 44, 45], In alignment with these reports, our experimental data revealed that GALNT3 overexpression significantly suppressed tMCAO/R‐induced phosphorylation of NF‐κB p65 in vivo (Figure S3A). Moreover, in OGD/R‐treated microglia, the inhibitory effect of GALNT3 on p65 activation was abolished upon TREM2 knockdown (Figure S3B). These findings collectively suggested that NF‐κB signaling may serves as a key downstream effector of the GALNT3/TREM2 axis in regulating microglial polarization and inflammatory responses during CIRI.

Beyond elucidating the downstream effects of GALNT3, our study also raises the question of why GALNT3 expression is suppressed under ischemic conditions. Based on the pathological process of CIRI, we speculate that its downregulation may be related to oxidative stress‐mediated transcriptional suppression. The occurrence of CIRI is typically accompanied by oxidative stress and excessive ROS generation. Oxidative stress has been shown to reduce the DNA‐binding activity of key transcription factors such as AP‐1, along with downregulating c‐Fos and c‐Jun protein expression [46, 47]. Given that these transcription factors are known to regulate genes involved in glycosylation and inflammatory responses, it is plausible that oxidative stress during CIRI inhibits GALNT3 transcription through similar mechanisms. Future studies investigating the transcriptional regulation of GALNT3 under ischemic stress will help validate this hypothesis and further clarify the upstream events in this pathway. In addition, although we recognize that incorporating human brain tissue data would significantly strengthen the translational relevance of our findings, the acquisition of such samples from patients with acute cerebral ischemia poses substantial ethical, logistical, and practical challenges, including the need for invasive procedures and stringent regulatory approvals. In future work, we will actively pursue clinical translation by evaluating the therapeutic potential of modulating the GALNT3/TREM2 axis in relevant patient populations. Finally, our experimental observations were limited to the acute phase (24 h post‐reperfusion) of CIRI. In future studies, we plan to systematically investigate the temporal expression pattern of GALNT3 and its downstream signaling across extended reperfusion periods, which will help clarify whether GALNT3 exerts transient or sustained effects on neuroinflammation, microglial polarization, and neurological recovery, thereby better defining its potential as a time‐sensitive therapeutic target.

## 5 Conclusion

Our findings collectively demonstrate that GALNT3 exerts significant neuroprotective effects against cerebral ischemia–reperfusion injury by enhancing the O‐GalNAc glycosylation and protein stability of TREM2, predominantly at serine 147. This GALNT3‐mediated post‐translational modification suppresses microglial M1 polarization, reduces the release of pro‐inflammatory cytokines, and attenuates oxidative stress and neuronal apoptosis. The identified GALNT3/TREM2 regulatory axis provides a novel mechanistic insight into the modulation of neuroinflammation following ischemic stroke and highlights GALNT3‐targeted O‐glycosylation as a promising therapeutic strategy for mitigating reperfusion‐associated brain damage.

## Author Contributions

Xinyi Fei and Jinghui Yang designed the study and wrote the manuscript. Rui Zhang and Yu Zhang performed experiments and analyzed data. Xinyi Fei provided technical support and assisted with data interpretation. Shan Yu revised the manuscript. All authors reviewed and approved the final manuscript.

## Funding

The authors have nothing to report.

## Ethics Statement

All procedures involving animal experiments were performed with consent from the Ethics Committee of China‐Japan Union Hospital of Jilin University (Approval No. 2025383).

## Conflicts of Interest

The authors declare no conflicts of interest.

## Supporting information


**Data S1:** Supporting Information.

## Data Availability

The data that support the findings of this study are available from the corresponding author upon reasonable request.
