# Supplementary material for: GALNT3 Inhibits the Progression of Cerebral Ischemia–Reperfusion Injury by Stabilizing TREM2 via O‐GalNAc Glycosylation
Source: CNS Neurosci Ther. 2026 Mar 11;32(3):e70828. doi: 10.1002/cns.70828 (PMC13093832; doi:10.1002/cns.70828)
Supplement: Supplementary file 1 — Data S1: Supporting Information. [file CNS-32-e70828-s001.docx]

Supplementary materials

## Materials and methods

### *qPCR assay*

Briefly, total RNA was isolated using TRIzol reagent (BioTeke, China), following the supplier’s protocol, and the concentration was determined using the UV spectrophotometer (Thermo, USA). cDNA was obtained by reverse transcribing RNA with the All-in-One First-Strand SuperMix (Magen, China). qPCR was performed on the Pangaea 3 fluorescence quantifier (Aperbio, China) using the SYBR Green Kit (Solarbio, China). The relative expression levels were analyzed using the 2^– ΔΔ CT^ method and β-actin expression was used to normalize the data. The primer information is shown as below: GALNT3 (mus), forward 5’-CTTCTGGCAAGCCGTTTA-3′ and reverse 5’-TTCAGGTGGTCGGGTGT-3′; IL-6 (mus), forward 5’-TAACAGATAAGCTGGAGTC-3′ and reverse 5’-TAGGTTTGCCGAGTAGA-3′; TNF-α (mus), forward 5’-ACAGAAAGCATGATCCGCGA-3′ and reverse 5’-TTGCTACGACGTGGGCTAC-3′; GALNT3 (homo), forward 5’-CCACCACCAGTGTCATA-3′ and reverse 5’-TAGCATCATCCACCAAA-3′; IL-6 (homo), forward 5’-GTCCAGTTGCCTTCTCCC-3′ and reverse 5’-GCCTCTTTGCTGCTTTCA-3′; TNF-α (homo), forward 5’-CGAGTGACAAGCCTGTAGCC-3′ and reverse 5’-TGAAGAGGACCTGGGAGTAGAT-3′.

### *Immunofluorescence staining*

Formaldehyde-fixed cells were incubated with 0.1% tritonX-100 (Beyotime, China) for 30 min and then blocked with BSA for 15 min. Cells were incubated with primary antibodies Tn antibody (1:100, Affinity, China), CD16/32 antibody (1:100, Proteintech, China) overnight at 4 °C. Cells were washed 3 times in PBS, then incubated with Cy3–conjugated goat anti-mouse IgG (1: 200, Proteintech, China) or Cy3–conjugated goat anti-rat IgG (1: 200, Abcam, UK) for 60 min. To stain cell nuclei, cells were incubated with DAPI (Aladdin, China). Images were obtained under the fluorescence microscope.

Paraffin-embedded tissue samples were cut into 5 μm thick sections. Sections were removed from paraffin with xylene and dehydrated with a series of graded ethanol. Samples were added to antigen repair solution and further incubated with BSA for 15 min at room temperature. Then sections incubation was done at 4 °C overnight with anti-Iba1 (1:200, Abcam, UK), anti-CD16/32 (1:100, Proteintech, China), anti-GALNT3 (1:100, CST, USA). Sections were further treated for 90 min with CY3 or FITC-conjugated IgG (1:200, Proteintech, China). Finally, sections were observed under the fluorescence microscope.

### *Determination of MDA, GSH/GSSG, and SOD*

Brain tissue was collected from mice. After rinsing and weighing, it was homogenized in 9 volumes of normal saline using a grinder. After centrifugation at 2500 rpm for 10 min, the supernatant was collected for determination. The activities of malondialdehyde (MDA), GSH/GSSG, and superoxide dismutase (SOD) were measured using assay kits (cat# A003, Nanjing Jiancheng, China), (cat# E-BC-K097-M, Elabscience, China), (cat# A001, Nanjing Jiancheng, China), respectively.

### *Enzyme-linked immunosorbent assay*

The brain tissue samples were added with 9 volumes of normal saline, centrifuged at 2500 rpm for 10 min, and the supernatant was taken for determination. Cell samples were centrifuged at 300×g for 10 min to remove sediment and then tested. The levels of IL-6 and TNF-α were determined using commercially available ELISA kits (Cat: EK206, EK106 (human); EK182, EK282 (mouse); Liankebio, China). The optical density was measured at 450 nm using a microplate reader (BIOTEK, USA).

### *Western blot assay*

The brain tissue and cell samples were harvested using RIPA buffer (Proteintech, China) and quantified by the BCA Protein assay kit (Proteintech, China). The SDS-PAGE (Proteintech, China) electrophoresis was used to detach the proteins, which were then transferred to PVDF (Thermo Fisher Scientific, USA). Next, the sealing solution (Proteintech, China) was used to block the membranes. Membranes were further immunoblotted with primary antibodies and secondary antibodies. Finally, the bands were visualized using ECL luminol (China, China). The primary antibodies including iNOS antibody (1:1000, Zenbio, China), GALNT3 antibody (1:2000, GeneTex, USA), TREM2 antibody (1:500, Santa Cruz, USA), NF-κB p65 antibody (1:10000, Zenbio, China), p-p65^(Ser536)^ antibody (1:10000, Zenbio, China). The secondary antibodies include goat anti-rabbit (1:10000, Proteintech, China) or goat anti-mouse (1:10000, Proteintech, China).

### *Fluoro-Jade C staining*

Degenerating neurons were evaluated by Fluoro-Jade C staining (Solarbio, China). In brief, brain sections were removed from paraffin with xylene and dehydrated with ethanol. Sections were incubated with potassium permanganate for 10min and Fluoro-Jade C was added for 20 min. Finally, images were captured with a fluorescence microscope.

### *DHE staining*

The OCT-embedded brain tissue samples were cut into slices 10 μm thick, rinsed with distilled water, then incubated with DHE reagent (1:100, Macklin, China) in dark light for 30 min. Slices were further added with an anti-fluorescence quencher. The staining effect was observed under the fluorescence microscope.

### *2, 3, 5-Triphenyl-2H-tetrazolium chloride (TTC) staining*

After model establishment, the brain tissue was collected and frozen at -20 °C for 2h. Brains were sliced into six consecutive sections and placed in 1% TTC solution at 37°C, and stained for 20 minutes in a dark environment. Slices were then removed for imaging and the infarct size was quantified.

## Figures


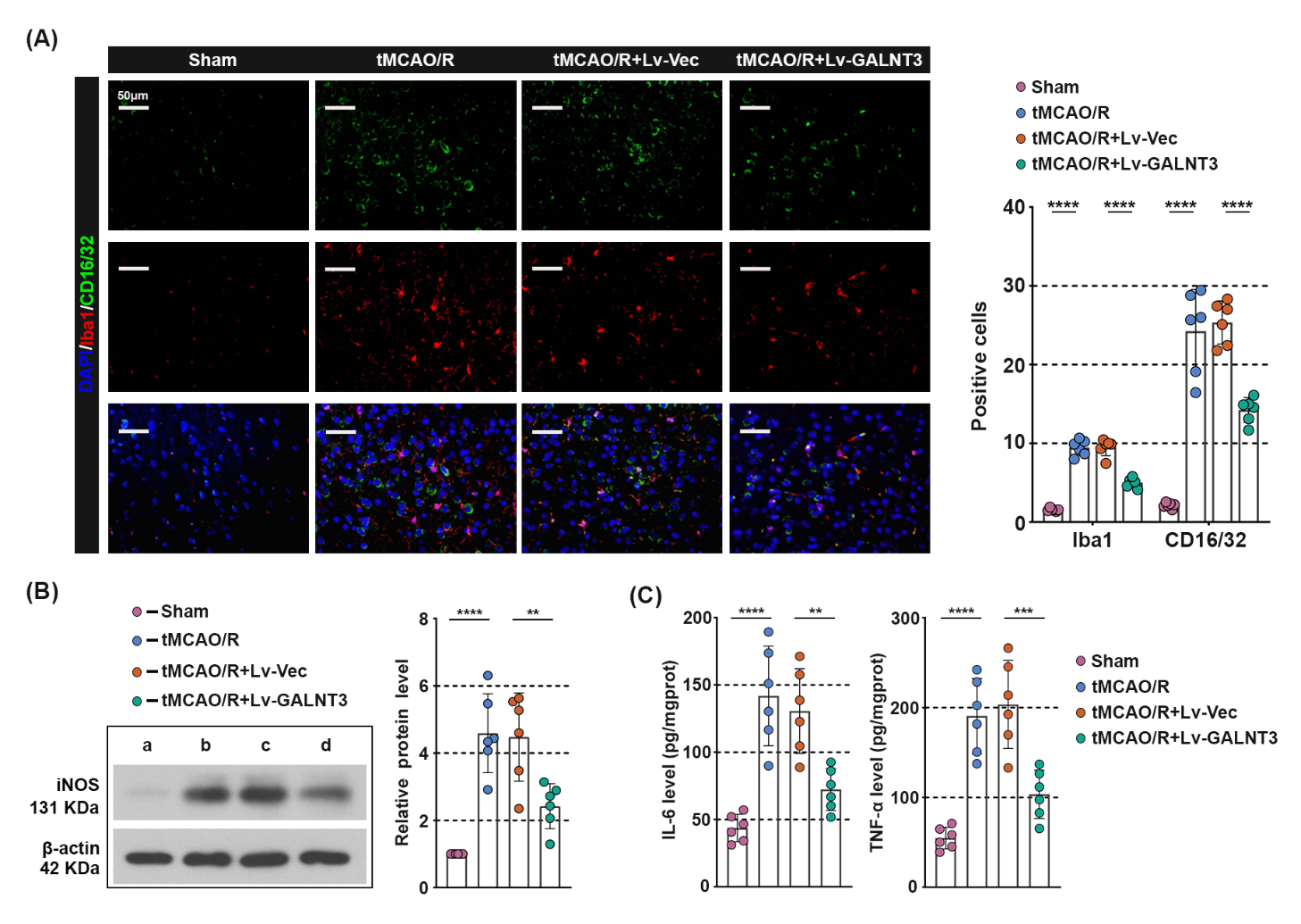


**Fig. S1** **GALNT3 inhibits inflammation and microglial M1 polarization in tMCAO/R mice.** (A) The coimmunostaining results of Iba1 (red) and CD16/32 (green) in the peri-infarct cortex. (scale bar, 50 μm). (B) The protein expression of iNOS in the peri-infarct cortex was detected by western blot. (C) The level of IL-6 and TNF-α was detected by ELISA. Data are shown as mean ± SD. n=6 per group. ^**^*p*<0.01, ^***^*p*<0.001, ^****^*p*<0.0001.


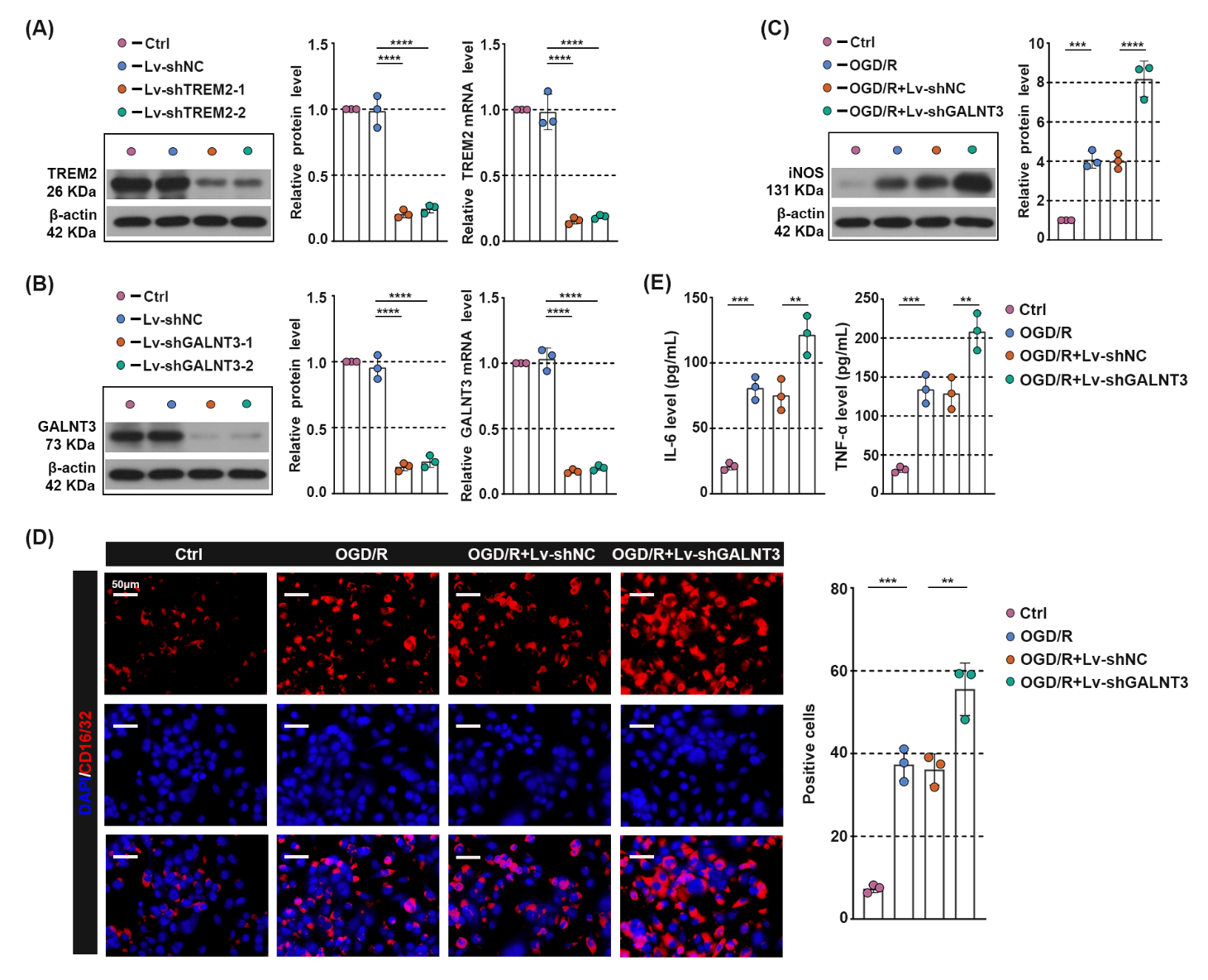


**Fig. S2 GALNT3 knockdown promotes inflammatory response and M1 polarization in OGD/R-induced microglia.** (A) The HMC-3 cells were infected with TREM2 knockdown or control lentivirus, and the expression of TREM2 was detected by qPCR and western blot after 48h. (B) The HMC-3 cells were infected with GALNT3 knockdown or control lentivirus, and the expression of GALNT3 was detected by qPCR and western blot after 48h. (C) Western blot analysis of iNOS in cells of each group. (D) The immunostaining results of CD16/32 in the cells. (Scale bar, 50 μm). (E) The level of IL-6 and TNF-α in the supernatant was detected by ELISA. Data are shown as mean ± SD. n=3 per group. ^**^*p*<0.01, ^***^*p*<0.001, ^****^*p*<0.0001.


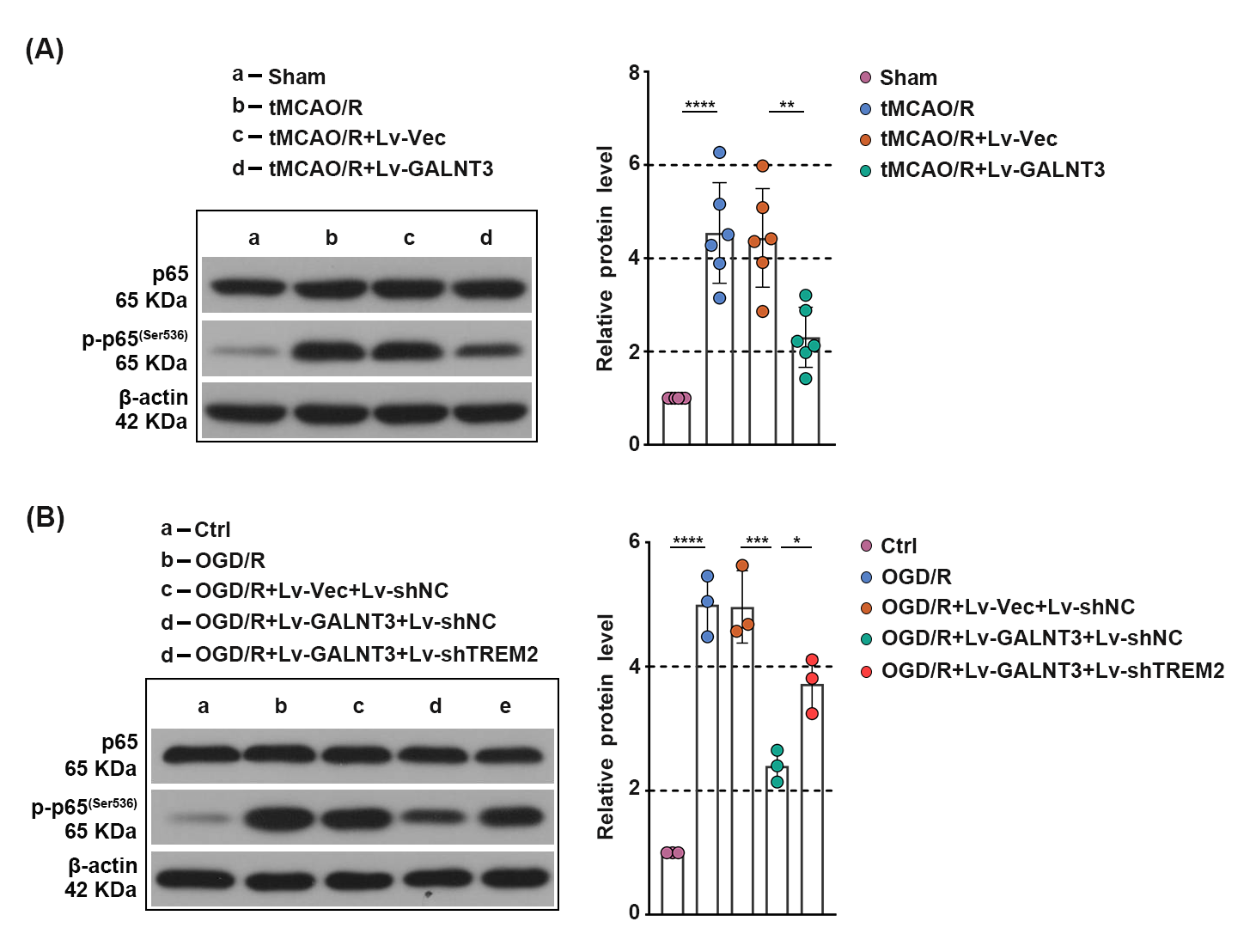


**Fig. S3 GALNT3 inhibits the activation of the NF-κB signaling pathway by regulating the expression of TREM2.** (A) Western blot analysis of NF-κB p65 andp-p65^（Ser536）^in the peri-infarct cortex. (B) Western blot analysis of NF-κB p65 andp-p65（Ser536 in cells of each group. Data are shown as mean ± SD. n=6/3 for animal or cell groups. ^**^*p*<0.01, ^***^*p*<0.001, ^****^*p*<0.0001.
